# Inverted Takotsubo Cardiomyopathy Induced by Dobutamine Stress Echocardiography with Atypical Presentation

**DOI:** 10.1155/2011/413645

**Published:** 2011-09-08

**Authors:** Christian Cadeddu, Silvio Nocco, Fabio Cadeddu, Martino Deidda, Pierpaolo Bassareo, Alessandra Serra, Mario Piga, Giuseppe Mercuro

**Affiliations:** ^1^Department of Cardiovascular and Neurological Sciences, University Hospital of Cagliari, Strada Statale 554, Km 4.500, Monserrato, 09042 Cagliari, Italy; ^2^Department of Nuclear Medicine, University Hospital of Cagliari, Strada Statale 554, Km 4.500, Monserrato, 09042 Cagliari, Italy

## Abstract

A 48-year-old woman was scheduled by our lab to perform a standard dobutamine/atropine stress echocardiogram. During the test, the patient referred to a slight chest discomfort and developed a progressive left ventricle akinesia of all midbasal LV segments, thus mimicking a midbasal ballooning. ECG persisted without significant abnormalities and with no raise of Troponin I. Coronary angiography showed normal coronary arteries and ventriculography a severe EF reduction and apical hypercontractility. Echocardiography showed a progressive improvement with a complete recovery 48 hours later. This is a rare case of inverted takotsubo syndrome induced by dobutamine stress echocardiography that occurred with atypical presentation.

## 1. Introduction

Takotsubo cardiomyopathy is increasingly recognised as a syndrome, usually provoked by severe mental stress and associated with acute LV apical “ballooning” or dyskinesia [1]. Its clinical symptoms and signs resemble those of an acute coronary event in presence of normally appearing epicardial coronary arteries. Major criteria of this syndrome are (i) an echocardiogram showing decreased apical contractility with basal hyperkinesia and, occasionally, intraventricular pressure gradients, (ii) the absence of obstructive coronary disease or angiographic evidence of acute plaque rupture, (iii) new ECG abnormalities (ST-segment elevation and/or T-wave inversion) or modest elevation in cardiac troponin, and (iv) the absence of pheochromocytoma and myocarditis. Although the aetiology of Takotsubo syndrome remains obscure, the combination of severe anxiety and catecholamine release appears to be the major trigger [2]. Cases of transient cardiomyopathy of mid and basal myocardial segments have recently been described [3, 4].

We present a rare case of inverted Takotsubo syndrome induced by dobutamine stress echocardiography with atypical presentation.

## 2. Case Report

A 48-year-old female with a long history of atypical precordial chest pain was referred to our echolaboratory for a dobutamine/atropine stress echocardiogram (DSE). One month before she was admitted to a peripheral general hospital for prolonged chest discomfort precipitated by emotional stress.  At arrival, resting ECG showed no abnormalities with no raise in troponin I or in other markers of cardiac necrosis. Transthoracic echocardiography only showed a mild impairment of LV diastolic function. At discharge, a bicycle ergometric test demonstrated ST segment depression in the inferolateral leads; during the test the patient did not experience any chest pain. Therefore, the patient was scheduled to perform a diagnostic DSE.

At our lab, the patient was clinically stable but with an anxious personality profile. The patient interview revealed a negative family history for cardiovascular diseases and that she had undergone an early menopause (age 45 years) without hormonal replacement therapy. Risk factors for coronary artery disease included smoking and hypercholesterolaemia, but not overweight or suffering from hypertension or diabetes. The patient underwent a full cardiovascular assessment (including physical examination, ECG, and transthoracic echocardiography), which revealed no cardiovascular or pulmonary diseases. 

**Figure 1:**
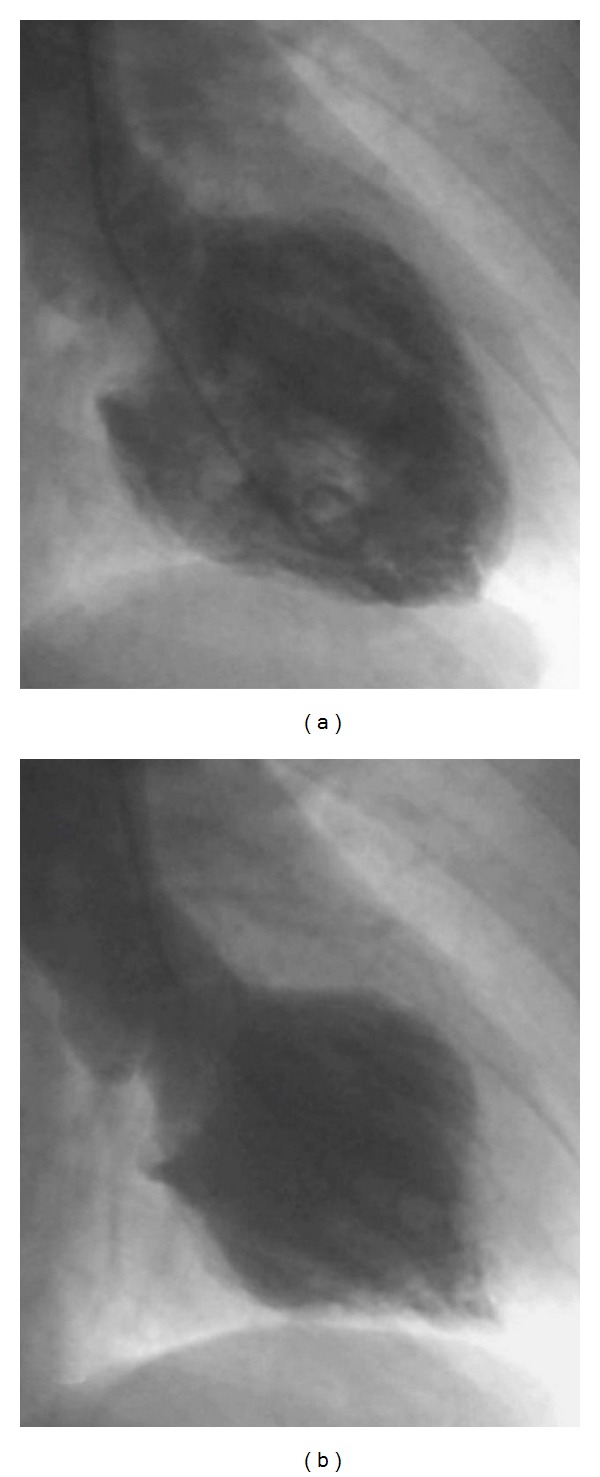
Ventriculography confirmed a global severe reduction of the systolic function (EF 28%) and apical hyperkinesis and severe dysfunction of the basal segments; (a) diastole, (b) systole.

A standard DSE protocol was used with 10 mcg/kg/min dose increments at 3 min intervals. Resting echocardiogram and blood pressure were normal. At 40 mcg/kg/min of dobutamine and following 0.5 mg of atropine (heart rate reached 154 bpm), we noticed anterior wall mid-basal hypokinesia without any ECG abnormalities. The test was interrupted and the recovery images showed a progressive worsening of the kinetic pattern with first marked systolic hypokinesia followed by akinesia of all LV mid-basal segments and severe impairment of global systolic function (ejection fraction 25%; Clip 1 (supplementary material) shows the 4 chamber echocardiographic view of the left ventricle demonstrating the severe impairment of global systolic function (supplementary material available on line at doi:10.1155/2011/413645)). ECG continued without showing any significant abnormalities and the patient referred to no chest pain. Blood pressure at this moment was 160/100, nitrates were administered i.v. and the patient was transferred to the cardiac catheterisation laboratory to perform a coronary angiography with suspected dobutamine-induced myocardial infarction. Ventriculography confirmed a severe reduction of the systolic function (EF 28%) and showed apical hyperkinesis and severe dysfunction of the midsegments with initial recovery of the basal segments (Figure 1); on the contrary, the angiographic study did not show any coronary lesions, despite a slow flow in the left coronary artery. The patient was then admitted to the intensive care unit, where the monitored plasma troponin I level remained in the normal range (<0.1 pg/mL) in the next 24 hours. Serial echocardiograms showed a progressive recovery of the systolic function with normal wall motion 48 hours later.

**Figure 2:**
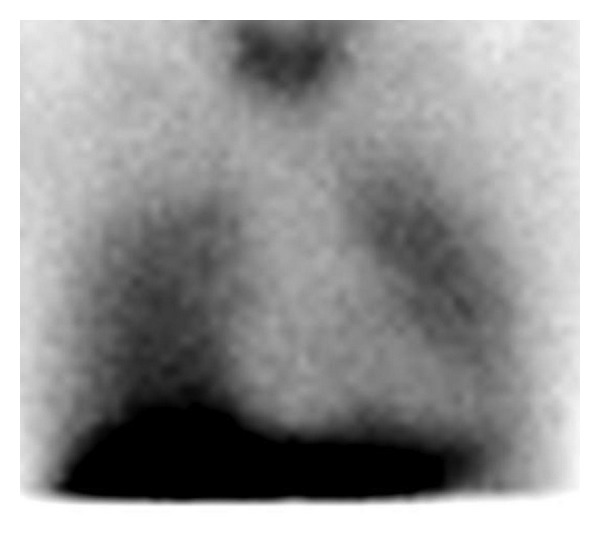
SPECT images showing a decreased myocardial ^123^I-MIBG uptake in the whole left ventricle.

One month later, the patient underwent a cardiac MRI study which confirmed the complete recovery of the systolic function and with no evidence of gadolinium late enhancement. Two months later, she underwent a ^123^I-Metaiodobenzylguanidine (MIBG) myocardial scintigraphy to verify the cardiac sympathetic activity. The early SPECT images showed a decreased myocardial ^123^I-MIBG uptake in the whole LV with an increased washout in the late images (Figure 2).

On this basis a *β*-blocker therapy was established for the patient. At 6-month followup the patient remained asymptomatic.

## 3. Discussion

This case report describes an episode of Takotsubo cardiomyopathy induced by DSE. Although transient cardiomyopathy induced by pharmacological stress has been reported previously [5], this is a rare case of dobutamine-induced inverted Takotsubo (mid-basal dysfunction), which occurred with an atypical presentation. Atypical features included no chest pain, no ECG abnormalities, and a lack of increase in cardiac troponin.

Several aetiological factors have been proposed for Takotsubo cardiomyopathy, including microvascular dysfunction, multivessel epicardial coronary spasm, catecholamine cardiotoxicity, and neurogenic stunned myocardium [6]. One or more of these pathogenetic interpretations conform to our patient's physical and clinical profile: an anxious, perimenopausal female who was a heavy smoker.

Furthermore, our patient suits the profile of an X syndrome: a postmenopausal woman with ECG changes during exercise, but without patent coronary stenosis. Consistent with this, an impaired endothelial function has been demonstrated in peri- and postmenopausal women. In comparison with premenopausal individuals of the same age, postmenopausal women showed increased vascular resistance, worse vasodilator reserve, and higher basal and stimulated plasma norepinephrine levels [7]. Moreover, reduced estrogen levels following menopause might be involved both by indirect action on the nervous system and by direct action on the heart [8].

In addition, several studies reported that Takotsubo cardiomyopathy may be accompanied by coronary microcirculatory abnormalities [9], which in turn correlate with the severity of myonecrosis and ECG abnormalities. 

The most obvious clinical finding is, however, that the Takotsubo syndrome has a predilection for women and shows a clear tendency to occur at times of very intense emotional stress, thereby suggesting a sympathetic-based aetiology. The close relationship with a high dobutamine dose, which was also observed elsewhere, supports a catecholamine-related mechanism in our patient.

The reduction of ^123^I-MIBG uptake agrees with the results of a study conducted with the same technique in 8 patients in which it was objectively documented that the Takotsubo syndrome is produced by an impaired cardiac adrenergic activity [6]. In that study, the dysfunction was present in the acute phase, whereas the ^123^I-MIBG scintigraphy documented a gradual normalization of sympathetic function after 3 months. Conversely, in the woman we studied, a global and severe uptake reduction was still present after the same time. This finding suggests that in some cases, like ours, the disturbance of cardiac innervation may be structural and, at least in part, independent of the ultimate cause triggering neurogenic myocardial stunning.

In conclusion, the most recent data on the atypical Takotsubo, given the variety of clinical profiles described in these patients [10], suggest the need for further research regarding the definition and pathophysiology of stress induced cardiomyopathy.

## Supplementary Material

Clip 1. 4 chamber view showing akinesia of all mid-basal segments of the left ventricle and severe impairment of global systolic function.

## References

[1] Sharkey SW, Lesser JR, Zenovich AG (2005). Acute and reversible cardiomyopathy provoked by stress in women from the United States. *Circulation*.

[2] Silberbauer J, Hong P, Lloyd GW (2008). Takotsubo cardiomyopathy (left ventricular ballooning syndrome) induced during dobutamine stress echocardiography. *European Journal of Echocardiography*.

[3] Hurst RT, Askew JW, Reuss CS (2006). Transient midventricular ballooning ayndrome. A new variant. *Journal of the American College of Cardiology*.

[4] Sanchez-Recalde A, Iborra C, Costero O (2009). Takotsubo cardiomyopathy—a new variant and widening disease spectrum. “Inverted Takotsubo” pattern related to catecholamine-toxicity. *International Journal of Cardiology*.

[5] Abraham J, Mudd JO, Kapur N, Klein K, Champion HC, Wittstein IS (2009). Stress cardiomyopathy after intravenous administration of catecholamines and beta-receptor agonists. *Journal of the American College of Cardiology*.

[6] Akashi YJ, Nakazawa K, Sakakibara M, Miyake F, Musha H, Sasaka K (2004). 123I-MIBG myocardial scintigraphy in patients with “takotsubo” cardiomyopathy. *Journal of Nuclear Medicine*.

[7] Mercuro G, Longu G, Zoncu S, Cherchi A (1999). Impaired forearm blood flow and vasodilator reserve in healthy postmenopausal women. *American Heart Journal*.

[8] Ueyama T, Kasamatsu K, Hano T, Tsuruo Y, Ishikura F (2008). Catecholamines and estrogen are involved in the pathogenesis of emotional stress-induced acute heart attack. *Annals of the New York Academy of Sciences*.

[9] Yoshida T, Hibino T, Kako N (2007). A pathophysiologic study of tako-tsubo cardiomyopathy with F-18 fluorodeoxyglucose positron emission tomography. *European Heart Journal*.

[10] Abdelmoneim SS, Mankad SV, Bernier M (2009). Microvascular function in takotsubo cardiomyopathy with contrast echocardiography: prospective evaluation and review of literature. *Journal of the American Society of Echocardiography*.

